# Leveraging homologous recombination repair deficiency in sarcoma

**DOI:** 10.15252/emmm.202317453

**Published:** 2023-03-17

**Authors:** Dea Slade, Joanna I Loizou

**Affiliations:** ^1^ Department of Medical Biochemistry, Max Perutz Labs, Vienna Biocenter Medical University of Vienna Vienna Austria; ^2^ Department of Radiation Oncology Medical University of Vienna Vienna Austria; ^3^ Comprehensive Cancer Center Medical University of Vienna Vienna Austria; ^4^ Center for Cancer Research, Comprehensive Cancer Centre Medical University of Vienna Vienna Austria

**Keywords:** Cancer, Chromatin, Transcription & Genomics, DNA Replication, Recombination & Repair

## Abstract

Personalised oncology is at the forefront of cancer research. The goal of personalised oncology is to selectively kill cancer cells while minimising side effects on normal tissue. This can be achieved by identifying and targeting cancer vulnerabilities that distinguish it from normal cells. Many cancers are deficient in high‐fidelity DNA repair pathways that maintain genomic stability, such as homologous recombination (HR). Such cancers are highly sensitive to targeted therapies that induce DNA damage or inhibit DNA repair pathways. A notable example and a poster child of personalised oncology are PARP1/2 inhibitors (PARPi) that selectively kill HR‐deficient (HRD) cancer cells by preventing repair of DNA gaps or single‐strand breaks (SSBs) (Slade, 2020). Inhibitors of cell cycle checkpoints such as CHK1 and WEE1 can also eliminate HRD cancers by pushing cancer cells through the cell cycle despite unrepaired DNA damage and causing death by mitotic catastrophe (Groelly *et al*, 2022). PARPi have been approved for the treatment of ovarian, breast, pancreatic, and prostate cancer but other cancer types with an HRD signature (HRD*ness*) may also respond to PARPi treatment. Planas‐Paz *et al* (2023) now show that many sarcomas show HRD*ness* and respond to PARP1/2 and WEE1 inhibitors, thus offering a new personalised oncology approach for this treatment‐refractory cancer.

## Graphical abstract

D. Slade and J. Loizou discuss the recent study by L. Planas‐Paz *et al* (in this issue of *EMBO Mol Med*) that identifies HRDness in sarcoma subtypes, and further demonstrates the clinical benefit of combining DNA damaging agents and inhibitors of DNA repair pathways in these subtypes.

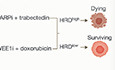

Sarcomas are a very heterogenous group of rare cancers that originate in the soft tissue of the body and in bone. Early‐diagnosed sarcomas have a good prognosis after resection and radio‐ or chemotherapy, whereas metastatic sarcomas are often unresectable and resistant to chemotherapy with a poor survival (Gómez & Tsagozis, 2020). A hallmark of many sarcomas is genomic instability caused by chromosomal copy number alterations and mutations in tumour suppressor genes such as *TP53*. Mutations in the HR genes *BRCA1* and *BRCA2* and mutational signatures of *BRCA* deficiency were previously identified in soft tissue and bone sarcoma patients, which provided the first indication of HRD*ness* in sarcomas (Kovac *et al*, 2015; Seligson *et al*, 2019; Li *et al*, 2020). Importantly, HRD*ness* extends beyond *BRCA1/2* mutations to include other HR genes such as *ATM*, *BARD1*, *CHEK1*, *CHEK2*, *PALB2*, *RAD51*, and *FANCL*. Therefore, there is a need to develop integrated approaches that can be used to identify HRD*ness* in cancers based on mutational, genomic, transcriptional, and cytological signatures (Slade, 2020).

**Figure 1:**
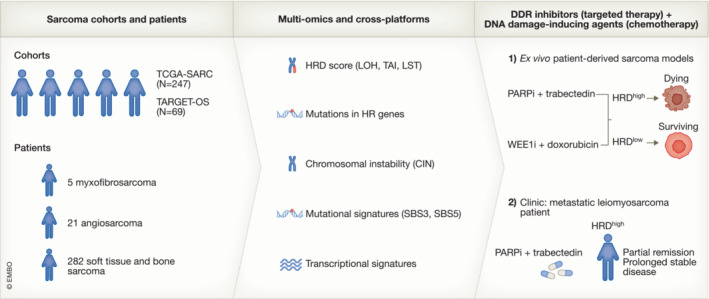
Towards a personalised oncological approach for sarcoma patients based on HRDness Soft tissue and bone sarcoma cohorts and patients were molecularly analysed on multi‐omics and cross‐platforms for the following characteristics to classify them as HRD^high^ or HRD^low^: HRD score based on loss of heterozygosity (LOH), telomeric allelic imbalance (TAI), and large‐scale transitions (LST); mutations in HR genes; chromosomal instability (CIN); mutational signatures of HRD (SBS3 and SBS5); transcriptional signatures. Based on the HRD score, (1) *ex vivo* models of HRD^high^ and HRD^low^ were established and treated with DNA damage response inhibitors (PARPi and WEE1i) in combination with chemotherapy drugs (trabectedin and doxorubicin), and (2) a treatment‐refractory metastatic leiomyosarcoma patient was selected and successfully treated with PARPi and trabectedin.

In this manuscript, Planas‐Paz *et al* (2023) develop a personalised oncological approach based on a large‐scale bioinformatics and multi‐omics platform to analyse various features of *HRDness* in publicly available sarcoma databases and sarcoma patient samples (Fig. 1). Specifically, they investigated (i) genomic signatures of HRD that define the HRD score (loss of heterozygosity [LOH], telomeric allelic imbalance [TAI] and large‐scale transitions [LST]), (ii) somatic mutational landscape of HR genes, (iii) chromosomal instability (CIN), including chromosomal rearrangements, amplifications and deletions, (iv) mutational signatures of HRD (small deletions, insertions and rearrangements), and (v) transcriptional signatures of DNA repair genes. Their cancer samples included TCGA‐SARC (sarcoma) cohorts (247 cases) and TARGET‐OS (osteosarcoma) cohorts (69 cases), as well as five myxofibrosarcoma patients, 21 angiosarcoma patients and 282 soft tissue and bone sarcoma patients. Based on this comprehensive analysis, Planas‐Paz *et al* (2023) demonstrate HRD*ness* as a general feature of sarcomas. They report that *BRCA2* and *FANC* genes are the most frequently mutated HR genes in sarcomas and find that a high HRD score is associated with HR gene mutations, increased HR gene expression and CIN.

To test whether HRD*ness* can be used as a predictive biomarker for PARPi sensitivity in sarcomas, as previously shown for ovarian, breast, pancreatic, and prostate cancers (Nguyen *et al*, 2020), Planas‐Paz *et al* (2023) established patient‐derived sarcoma cell models with high or low HRD scores and tested their sensitivity to PARPi (olaparib and niraparib) and WEE1i (adavosertib) (Slade, 2020; Groelly *et al*, 2022). HRD^high^ samples showed impaired RAD51 foci formation, in accordance with defective HR. HRD^high^ samples were more sensitive to PARPi and WEE1i compared to HRD^low^ samples. Alone, chemotherapeutic DNA‐damaging agents commonly used in sarcoma therapy such as oxaliplatin (crosslinking agent), doxorubicin (topoisomerase II inhibitor), and trabectedin (alkylating agent) did not show differential effects relative to the HRD score. However, trabectedin and doxorubicin showed synergistic effects when combined with PARPi or WEE1i, respectively, in HRD^high^ samples, which was further exploited in a clinical setting.

By treating a metastatic leiomyosarcoma patient with the PARPi olaparib combined with trabectedin, Planas‐Paz *et al* (2023) report that this led to partial remission and prolonged stable disease over a period of 18 months. This compelling proof of concept, along with ongoing clinical trials that are testing the benefits of combining targeted and chemotherapy in sarcomas, will undoubtedly instigate future clinical trials in which sarcoma patients should be stratified based on HRD as a biomarker guiding the choice of therapy.

An important aspect to consider for clinical applications of PARPi in sarcomas is the fact that a proportion of these may develop resistance due to a range of mechanisms including restoration of wild‐type *BRCA* through secondary mutations, inactivation of the non‐homologous end‐joining pathway (NHEJ) due to *TP53BP1* and *SHLD1* mutations, concomitant induction of alternative end‐joining (alt‐EJ), and upregulation of efflux pumps (Dias *et al*, 2021). Thus, combinatorial treatments with other DNA damage response inhibitors that target, for example, ATR, a kinase that signals replication stress, or POLθ, the helicase and polymerase that functions in alt‐EJ, have recently emerged as a promising strategy to overcome PARPi resistance in the clinic (Schrempf *et al*, 2021).

Overall, the study by Planas‐Paz *et al* (2023) represents an important step in addressing an unmet need in advanced and refractory sarcomas by identifying HRD*ness* as a predictive biomarker for the efficiency of a combinatorial treatment strategy of PARPi and trabectedin or WEE1i and doxorubicin. Ongoing clinical trials (https://clinicaltrials.gov) with PARPi (olaparib/niraparib/talazoparib) alone or in combination with trabectedin, temozolomide (methylating agent), irinotecan (topoisomerase I inhibitor), POLθ inhibitors, ATR inhibitors or immune checkpoint inhibitors (durvalumab/dostarlimab), amongst others, will further probe the efficacy of PARP inhibitors in advanced and refractory sarcomas.

## Author contributions

**Dea Slade:** Conceptualization; writing – original draft; writing – review and editing. **Joanna Loizou:** Writing – review and editing.
